# pH and Urea Estimation in Urine Samples using Single Fluorophore and Ratiometric Fluorescent Biosensors

**DOI:** 10.1038/s41598-017-06060-y

**Published:** 2017-07-19

**Authors:** Rashmi Chaudhari, Abhijeet Joshi, Rohit Srivastava

**Affiliations:** 1 Department of Biosciences and Bioengineering, Indian Institute of Technology Bombay, Powai, Mumbai, Maharashtra 400076 India; 2 Centre for Biosciences and Biomedical Engineering, Indian Institute of Technology Indore, Simrol, Indore, Madhya Pradesh 453552 India

## Abstract

Kidney diseases remain often undiagnosed due to inefficient screening methods available at patient’s disposal. Early diagnosis and effective management of kidney problems can best be addressed by the development of biosensors for commonly occurring clinical biomarkers. Here we report the development of single fluorophore and dual fluorophore ratiometric biosensors based on alginate microspheres for pH and urea analysis in urine samples. A facile method of air driven atomization was used for developing these polymeric fluorophore and enzyme based biosensors. Ratiometric biosensors were developed using layer-by-layer coating of polyelectrolyte conjugated to reference fluorophores. Biosensing studies using these biosensors showed that samples in pathophysiological range can be measured having pH range of 4–8 and urea levels between 0–50 mM. Testing of urine samples using these biosensors showed that both pH and urea detection can be accurately performed without interference. Thus, we believe that FITC-Dextran and FITC-Dextran/RuBpy based pH and urea biosensors show a great potential to be translated as a point of care device for pH and urea biosensing in early detection and continuous monitoring of kidney diseases.

## Introduction

Kidney ailments form the 9^th^ leading cause of mortality in United States with a global prevalence of 10–26%. The prevalence of kidney diseases can be reduced by deploying biomarker screening strategies. Preliminary clinical status of patient with kidney ailments is established by measurement of physiological parameters and biomarkers like glomerular filteration rate, urea, creatinine, pH, glucose etc. in blood or urine. pH and urea estimation is a routine clinical investigation performed to assess kidney functioning. Literature cites several detection methods for pH and urea based on amperometric and spectrophotometric methods^1–8^. Advancements in biosensor technology have led way to convert detection methods into point-of-care devices. Gas selective electrodes, pH electrodes, ammonium ion selective electrodes, CO_2_ gas electrode, etc. are some of the methods employed for pH and urea detection. Commercially available amperometric biosensors of pH and urea estimation including the flow injection based analysis systems are accurate but costly, complicated, are prone to interferences in biological fluids and lack point-of-care measurement capability^9^. Fluorescence based pH detection typically involves use of single or combination of pH responsive fluorophores where as urea detection uses indirect determination based on ammonium and hydroxide ion detection after urease catalytic reaction. Optical enzyme based fluorescent biosensors employing single or combination of fluorophores acting as indicator and reference fluorophores, can prove to be superior when specificity, sensitivity and amenability of developing point-of-care measurement systems is compared.

Single fluorophore (non-ratiometric) based estimation of pH has beed described by several researchers using fluorophores like rhodamine, heptamethine cyanine-based dye, 6-carboxyfluorescein, 2′,7′-bis-(2-carboxyethyl)-5-(and-6)-carboxyfluorescein, 1,4- dihydroxyphthalonitrile, 8-hydroxypyrene -1,3,6- trisulfonic acid (HPTS), seminaphthorhodafluor (SNARF)/seminaphthofluorescein (SNAFL) dyes, boron-dipyrromethene (KBH-01), etc. BODIPY based fluorphores have also been utilized for pH sensing due to their high molar absorptivity, fluorescence properties, narrow emission peaks and high stability^10^. A Rhodamine based fluorescent sensor developed by Tian *et al*., showed increase in fluorescence below neutral pH in the range 1 to 4 between 450–650 nm^11^. Wu *et al*. developed a heptamethine cyanine-methylpiperazine near-infrared sensor which showed high sensitivity towards pH in aqueous solutions with a 7 fold enhancement from pH 9.8 to 2.43 in fluorescence intensity with a large stokes shift (>100 nm)^12^. Apart from the fluorophores, quantum dots (ZnS) have been also used for detection of pH and urea, however the range of detection of urea has been found to be upto 4 mM which does not cover pathological concentrations of urea in blood or urine^13^. Non-ratiometric/single fluorophore and urease based estimation of urea has also been described in the literature. Octadecyl nile blue and nonactin based biosensor for urea described by Wolfbeis *et al*. showed a linear range of 0.01–1 mM of urea^14^. Xie *et al*. showed a coupling of pH indicator trisodium 8-hydroxypyrene-1,3,6-trisulphonate in fibre optic membranes to measure in a linear range of 0–5 mM with a response time of 15 min in steady state^15, 16^. Use of single fluorophores for biosensor development is subject to limitations like instrumental variations, fluorophore concentration and distributions, inner filter effects, variations in distances between sample and excitation source or detector and environmental variations. These perils of single fluorophore based estimation can be removed by ratiometric estimation using a reference fluorophore. Ratiometric estimation of several analytes like Superoxide (O_2_^−^)^17^, hydrazine^18^, Copper^19^, NO^20^, Oligonucleotides^21^, Fluoride^22^, GSH^23^, Mercury^24, 25^ and Phosphate^26^ have been described. Ratiometric estimation of pH using Carboxynaphthofluorescein (CNF) and ETH 5350 which have two peaks and are pH sensitive can be used for ratiometric measurement. Co-immobilized silica matrices containing dual fluorophores like Fluorescien and phenosafranine have been used for pH determination in water samples by Gao *et al*.^27^ Ke *et al*. described synthesis of FITC-BSA/Gold nanoclusters (AuNCs) which act as single wavelength excitation based indicator (I_525_/I_670_). The indicator system was used for ratiometric pH detection based systems were AuNCs acted as internal standard. The pH response was found to be in the range of 5–8.5 with an interval of 0.1 units and a regression coefficienct of 0.9905^28^. Liu *et al*. have synthesized porphyrin derivatives for ratiometric fluorescent pH sensors having intensity ratios at 656/719 nm to obtain sigmoidal relationship at an excitation wavelength of 446 nm^29^. Other fluorescent ratiometric biosensors developed by Niu *et al*. showed its response can be used to detect extremely acidic pH. Other examples of ratiometric pH sensing include fluorophores and wavenlengths like porphyrins (I_446_/I_418_), 1,1-dimethyl-2-[2-(quinolin-4-yl)vinyl]-1H-benzo[e]indole (QVBI) (I_522_/I_630_), NIR-Benzothiazole (I_748_/I_672_), Fe_3_O_4_ nanocrystals-MMP-9- N-carboxyhexyl derivative of 3-amino-1, 2, 4- triazole-fused 1,8-naphthalimide (ANNA) (I_748_/I_672_), SNARF-1-dextran (I_580_/I_640_), carbon dots, quantum dots, bacteriophage particles^30^ etc. Fluorescein tagged aptamer sensors based on silicon nanodots have been described for *in vivo* pH measurement using cancer cell lines^31^. *In vivo* imaging and detection of acidic pH has been peformed using croconaine rotaxane dye which acts as a pH sensitive, near infra red ratiometric dye^32^. Quantum dots along with dye can also function as ratiometric sensors where in the estimation is based on dual excitation-single emission, dual excitation-dual emission or single excitation-dual emission^33^. Urea estimation using ratiometric fluorescent biosensors are meagre. Oxazine 170 perchlorate (O17)-ethyl cellulose (EC) membrane (I_630_/I_565_) developed by Duong *et al*. for biological urine samples^34^. FITC-BSA/AuNCs system developed by Ke *et al*. described earlier was also used investigated as a ratiometric urea sensor. FITC-BSA/AuNCs sensor showed a linear range of 0–12 mM with a LOD of 0.19 mM with an incubation time of 30 min. Tsai and Doong employed FITC-dextran and TRITC-dextran in sol-gel array displaying an improved detection range of 0–10 mM after incubation of 15 min^35, 36^. Kazakova *et al*. showed encapsulation of SNARF-1 (I_580_/I_640_) and urease in polyelectrolyte microcapsules developed from CaCO_3_ to achieve urea biosensing in the range of 10^−6^ to 10^−1^ M. Fluorescent urea measuring systems in the literature either do not cover physiological and pathological concentrations in serum or face limitations of a higher response times^37^.

The current research reports development and testing of Fluorescein-iso-thiocyanate-dextran (FD)/FD-Urease encapsulated alginate microcarriers as single/ratiometric fluorophore based fluorescent biosensors for detecting pH and urea in urine samples. Layer-by-layer self-assembly of polyallyl amine hydrochloride (PAH) and polystyrene sulfonate (PSS) in conjunction with Tris(bipyridine)ruthenium(II) chloride (RuBpy) over FD/FD-Urease loaded alginate microspheres aids in developing ratiometric sensors for pH and urea. FD and RuBpy function as indicator and reference fluorophores, respectively. The developed biosensors were characterized using optical microscopy, zeta potential, SEM, CLSM and encapsulation efficiency and validated for accuracy, response time, linearity, range, intra-day/inter-day reproducibility, stability and performance in urine samples.

## Results and Discussion

### Development and Characterization of Single Fluorophore/LBL assembeled Ratiometric pH and Urea Biosensor

**Figure 1:**
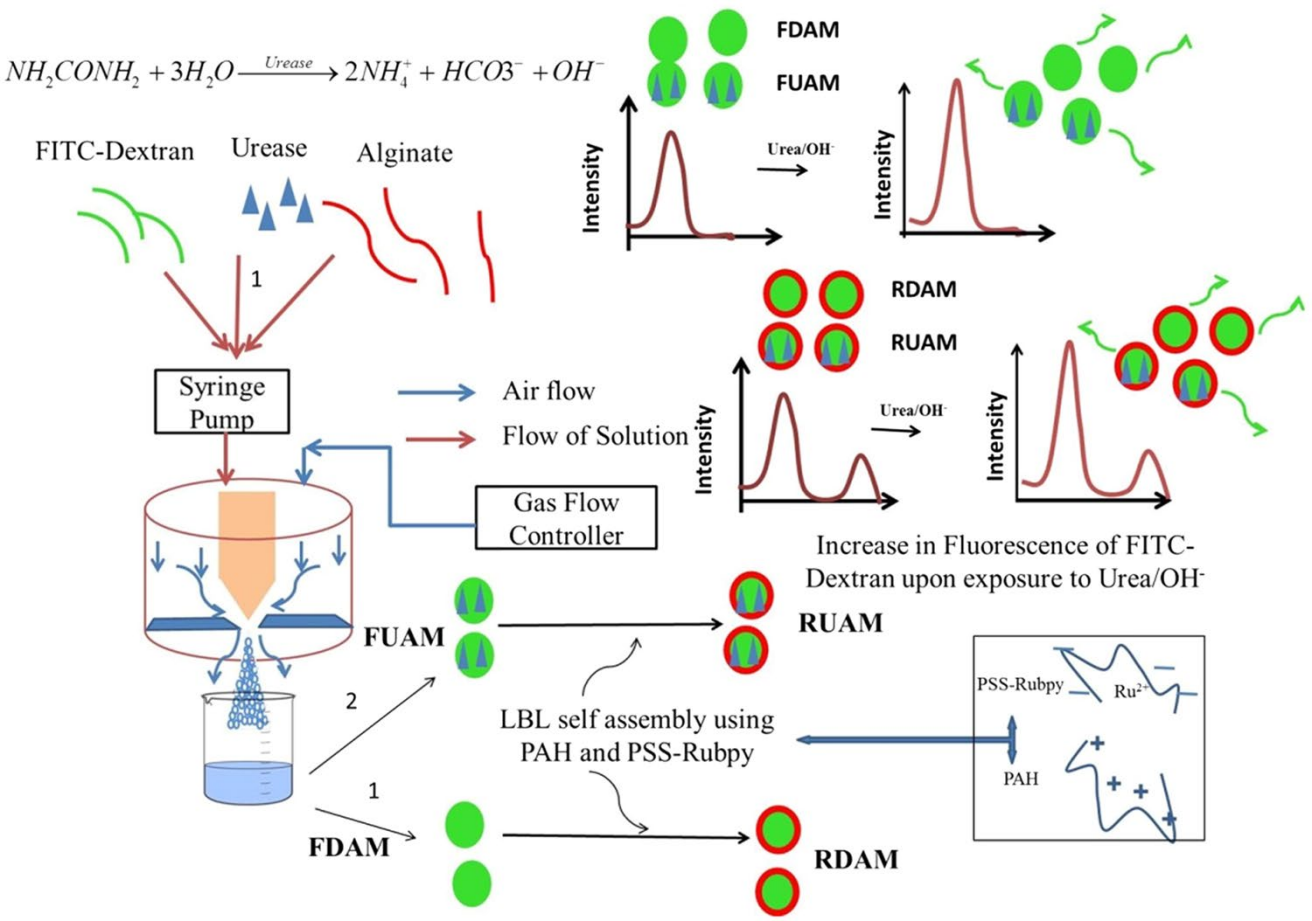
Schematic of development and mechanism of single fluorophore/ratiometric pH (Scheme 1) and urea biosensor (Scheme 2).

Sensor performance of enzyme based sensors is critically dependent on matrices used for encapsulation. Alginate has remained a material of choice for enzyme encapsulation in the recent past owing to its mild gelation without use of any toxic chemicals^38–40^. FD loaded alginate microspheres (FDAM) have been developed using air driven atomization and crosslinking with CaCl_2_ to form single fluorophore pH biosensor. Negatively charged FITC is fluorescent in nature in comparison to non-ionic and cationic non-fluorescent counterparts. pH induced ionization determines the varied fluorescent response of FITC, for e.g. acidic pH leads to higher protonation of FITC causing a reduced fluorescence emission and an alkaline pH causes an increase in fluorescence emission. FD (500 kDa) shows a superior stability and similar sensitivity upon encapsulation in comparison to low molecular weight FD’s due to lower tendency of leaching from the matrix (Figure S1). Development and optimization of alginate microspheres containing various biomolecules employing air driven atomization method has beed described in earlier reports^39, 41–45^. Similarly for development of a single fluorophore based urea biosensor involved co-immobilization of urease along with FD to form FD-Urease loaded alginate microspheres (FUAM) (Fig. 1, Scheme 1). Urease (Mol. Wt: 480 kDa) was used as a biological recongnition element which catalyzes conversion of urea to ammonium and hydroxide ions. The alkalinity generated in the microenvironment due to the catalytic reaction causes an increase in fluorescence emission at 520 nm.

Urease and FD both being high molecular weight compounds affect the loading of each other in the alginate microcarriers due to similar encapsulation efficiencies at varied concentrations. Encapsulation efficiency of FD and urease as determined by UV-visible spectroscopy were found to be greater than 80% for all tested concentrations between 0.25–1 mg-ml^−1^ of FD and 1–4 mg-ml^−1^ for urease (Figure S2). A subsequent layer-by-layer (LBL) assembly of polyallyl amine hydrochlorde (PAH) and polystyrene sulphonate (PSS) forming PAH-PSS (RuBpy)-PAH over FDAM and FUAM, was used in developing a ratiometric pH sensor (RDAM) and a ratiometric urea sensor (RUAM), respectively. Owing to positive electrostatic charge of Ru^2+^ ions electrostatic attachment of PSS was used to stabilize the RuBpy molecules in the LBL coatings. Use of FD (λex: 488 nm and λem: 520 nm) and RuBpy (λex: 488 nm and λem: 610 nm) in a matrix enables a single excitation and dual emission ratiometric sensing.

**Figure 2:**
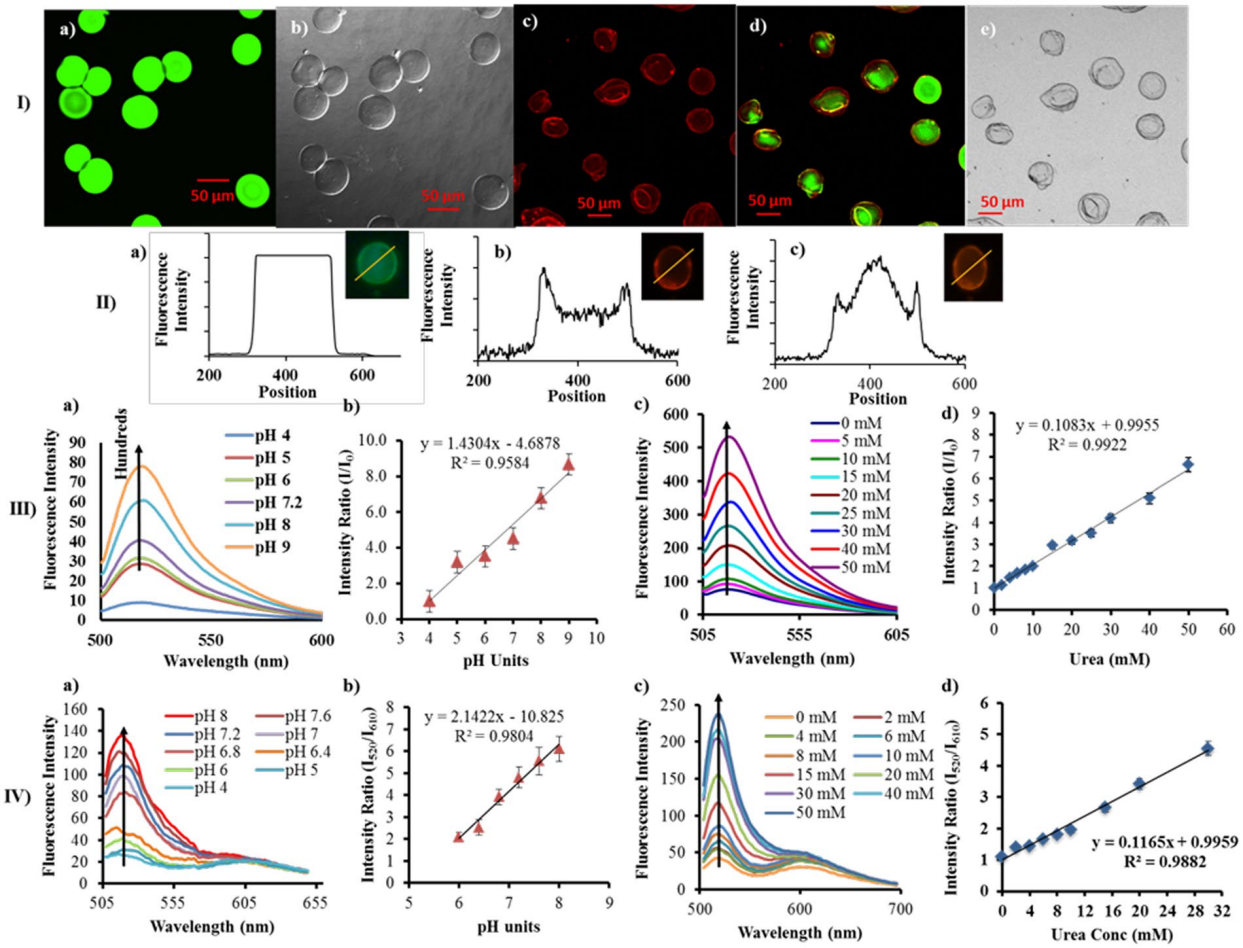
[**I**] Particle characterization of single fluorophore and ratiometric sensors using CLSM, and corresponding DIC images: (a,b) FDAM/FUAM, (c–e) RDAM/RUAM, [**II**] CLSM based line scan analysis of (a) FDAM/FUAM, (b) RuBpy loaded LBL assembled urease loaded alginate microspheres, (c) An overlay image of FD-RuBpy loaded microcarriers. [**III**] Single fluorophore based pH sensing (a,b) and urea sensing (c,d) and [**IV**] Ratiometric pH sensing (a,b) and urea sensing (c,d).

Particle size and morphological evaluation using optical microscopy for FDAM/FUAM and RDAM/RUAM indicated that uniform sized spherical microspheres can be formed and that FD and urease can be loaded into the microcarriers without strucutural deformation and maintaining sphericity (Figure S3). Distribution of fluorophores and microsphere characterization carried out by confocal laser scanning microscopic (CLSM) imaging showed FD is uniformly distributed inside the alginate microcarries (Fig. 2I(a,b)) in both FDAM and FUAM. The uniform distribution was further confirmed by intensity distribution profiles (Fig. 2IIa). CLSM imaging of RDAM, RUAM and corresponding DIC images proved the presence of RuBpy (red color) fluorophore coated over the FD containing microcarriers (green color) particle (Fig. 2I(c–e)). The CLSM based line scan analysis also confirmed the presence of both the fluorophores, FD in the core and RuBpy in the LBL coatings. The successful layer-by-layer self-assembly of PAH-PSS-PAH over alginate microcarriers have also been indicated by alternating zeta potential values described by Jayant *et al*.^39^ SEM images confirmed that the alginate microspheres are porous, spherical and LBL coated in nature visualized as different surface properties (Figure S3).

### Biosensing and Validation of pH and Urea Biosensors

A thorough evaluation of sensing components in terms of suitability, concentrations and stability was performed for developing these biosensors. The optimization process aimed at improving the sensor range, sensitivity to detect physiological and pathological concentrations, reducing the sample volume and response time. Encapsulation of fluorophore and enzyme together in the microspheres enables localization of sensing assay allowing for higher sensitivity and lower response time of biosensor in comparison to solution phase estimation. Fluorescence response of FITC upon exposure to different pH values (0–10 units) showed a typical sigmoid curve with a near linear response in the range of 4–8 pH units (Figure S1). An overlay of changes in fluorescence intensity at 520 nm in response to different pH and urea concentrations are described in Fig. 2III(a,c) for FDAM and FUAM respectively. The results indicate that exposure of alkaline pH either directly or indirectly in the form of catalytic reaction of urease both cause an increase in fluorescence intensity. FDAM and FUAM both showed a linearly increasing fluorescence response at 520 nm in response to increasing pH or urea concentrations (Fig. 2III(a–d). A linear regression analysis indicated that pH detection is possible in the range of pH 6–8 with a regression coefficient of 0.96, with 36.6% change in fluorescence intensity with 0.1 unit change in pH and a response time of 5 min (Fig. 2III(a,b)). Similarly, urea sensing using FUAM (Fig. 2III(c,d)) experiments suggested that urea detection is possible in the range of 0–50 mM with a regression coefficient of 0.992, sensitivity of 10.6% mM^−1^ and a response time of 8 min. Sensing studies involving RDAM and RUAM clearly indicated that FD functions as an indicator fluorophore (λem = 520 nm) and RuBpy functions as a reference fluorphore (λem = 610 nm). The results confirm the pH responsive nature of FD and pH insensitive nature of RuBpy (Fig. 2IVa–d) (Figure S4). Using these two fluorophores it is possible to transform FDAM/FUAM into RDAM/RUAM, respectively. RDAM showed and improved performance with 20% change in fluorescence intensity with 0.1 unit change in pH, in the range of 6–8 and a response time of 5 min (Fig. 2IVa,b). Using RUAM the sensitivity was found to be 10.5%/mM of urea in the linear range of 0–40 mM with a regression coefficient of 0.988 (Fig. 2IVc,d). Saturation of intensity ratios after 40 mM which occurs due to high pH in the microenvironment. Sensitivity of urea detection was increased upon encapsulation in microspheres in all the four formulations which occurs due to close proximity of urea catalysis and sensing assay. Similar sensitivity values of FUAM and RUAM suggest that LBL assembly of reference fluorophores does not affect adversely in the sensing performance. The range of detection of RUAM can be increased by increasing the number of sensor particles or by reducing the sample volume. A reduced sample volume containing urea concentration was more effective in increasing the range of detection upto 50 mM when compared with the solution phase sensing (Figure S5). Response time for FDAM/RDAM was found to be 5 minutes and for FUAM/RUAM it was found to be 8 minutes, respectively. LOD values of FDAM, RDAM, FUAM and RUAM were found to be 5.1, 5.3 pH units and 1.02, 0.85 mM of urea, respectively. The intra-day reproducibility for sensing assays within a day from a single batch for FUAM and RUAM urea sensors was found to be reproducible with a % RSD value of 3.5 and 2.6%. Inter-day reproducibility studies were carried out using a freshly prepared batch of urea biosensor on three consecutive days. The inter-day reproducibility indicated by % RSD value for sensor response tested on three consecutive days for FDAM, RDAM, FUAM and RUAM sensors of 7.5%, 6%, 5% and 3.8%, respectively. The % RSD values clearly suggest that the variability of detection of pH/urea was reduced in both the ratiometric sensors. Swati M. *et al*. also described similar ratiometric biosensors using urease and cresol red based biosensors developed using alginate microspheres^46^. Tsai *et al*. reported the use of FD and TRITC-Dextran in a sol gel based micro-array to measure urea levels in the range of 0–10 mM.

### Detection of pH and Urea in Urine Samples

**Figure 3:**
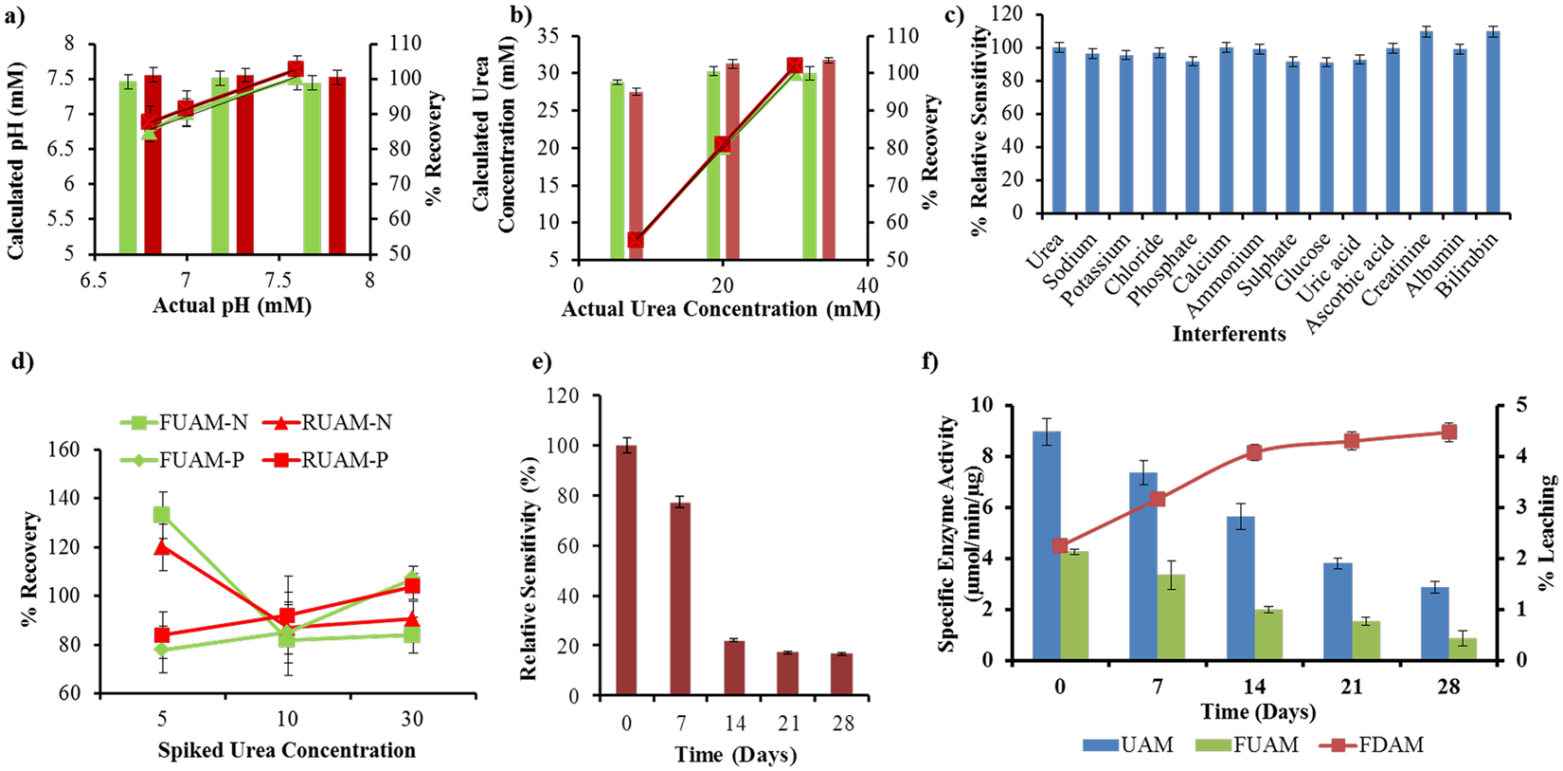
Evaluation of peformance of sensors: (**a**) Accuracy studies of pH sensors with standard pH samples, (**b**) Accuracy studies of urea sensors with different urea concentrations spiked in urine samples obtained from healthy volunteers (n = 3), (**c**) Interference Study for commonly present compounds in urine, (**d**) Accuracy studies of urea sensors with different urea concentrations spiked in urine samples obtained from kidney disease patients (n = 3), (**e**) Stability study and (**f**) Urease activity studies and leaching study of FD.

Accuracy studies performed by comparing unknown concentrations of pH and urea standard solutions in the range of detection and represented as % recovery show that FDAM and RDAM was found to be 98.9–100.3% and 100–101.2%, respectively standardized using a pH meter. Similarly, % recovery values for FUAM and RUAM were found to be 97.5–100.5% and 95–103.5%, respectively (Fig. 3a,b). The accuracy studies using standard solutions show that the detection of pH and urea is determined precisely. Regression coefficient for % recovery plot of actual concentration vs. calculated concentration for FDAM, RDAM, FUAM and RUAM was found to be 0.988, 0.999, 0.999 and 1.00 respectively (Table S2).

The values clearly indicate that the ratiometric sensors RDAM/RUAM provide a better regression coefficient for % recovery than FDAM/FUAM. pH measurement of urine solutions was directly determined by a pH meter and compared to values determined from the FDAM and RDAM biosensors. % recovery values for pH detection of urine samples using FDAM and RDAM was found to be 95.1% and 101.6%, respectively. Urea concentrations were spiked in 20X diluted urine to reduce errors in estimation due to inherently acidic pH of urine. % recovery of urea spiked urine solutions obtained from healthy volunteers were found to be 81.9–133% and 87–120% for FUAM and RUAM, respectively. When urine samples from kidney patients were tested for accuracy of determination the % recovery values were found to be in the range of 78–107% and 84–104%. The lower mean recovery values in case of urine samples from kidney disease patients may be due to higher concentrations of interferents in concentrated samples of urine or presence of drugs and their metabolites due to the therapy been undertaken by the patients. Ratiometric sensors were found to be superior than the single fluorophore sensors in both kinds of urine samples (Fig. 3d).

### Stability, Interference and Leaching Studies of pH and Urea Biosensors

Sensor performance was compared using relative percent sensitivity changes calculated from the slope values over a period of one month. FDAM and RDAM sensors show that the relative sensitvity values decrease to 80% at the end of 28 days indicating a fairly good stability upon storage in refrigerated conditions. FUAM and RUAM relative sensitivity values on the other hand change significantly with drop upto 22% in 14 days and 16% after 28 days which suggests both urea sensors can be used only for 7 days (Fig. 3e). A lower stability of urea sensors suggest that the enzyme activity is affected due to encapsulation along with FD. An effect of reduced activity and stability of 7 days was described by Saxena A in alginate gels^47^. An interference study using commonly occurring interfereing analytes in biological samples like glucose, uric acid, ascorbic acid, creatinine, sodium, potassium, calcium, ammonium, chloride, sulphate, phosphate, albumin and bilirubin in their maximum physiological concentrations along with urea standard solutions showed urease activity ≥90% (Fig. 3c). Buffers of different pH exert influence on the sensor performance of FUAM where in increasing the pH of the buffer reduces the analytical range in which the sensor is functional (Table S1). Urease leaching from FUAM using Bradford’s test showed minimal leaching (<5%) upto 28 days. Leaching of FD 500 kDa from FDAM and FUAM was found to be very less (<5%) over a period of one month (Fig. 3f). Leaching studies substantiate the dependence of molecular weight for physical stability of macromolecules in alginate matrix. FD 500 kDa and Urease 545 kDa owing to their similar molecular weight have similar leaching profiles from alginate microsphere matrix. Enzyme activity of urease was determined using Nessler’s reagent (Potassium-mercuric-iodide) which reacts with ammonium ions produced during urea catalytic reaction. Potassium mercuric iodide forms brown colour product on reaction with ammonia which gives an absorbance at 405 nm. Ammonium ions were quantified using a calibration curve prepared from ammonium chloride. Enzyme activity study aimed at understanding the effects of matrix and dye on urease like in UAM, FUAM for over a period of 28 days. Specific enzyme activity was determined using Bradford’s reagent maintaining the concentration of enzyme same in all the formulations. Solution phase specific urease activity was found to be 25.4 µmol min^−1^ µg^−1^ which reduces to 9.34 µmol min^−1^ µg^−1^ over a period of 28 days when stored at 4 °C. Urease entrapped in alginate microspheres (UAM) showed a residual activity of 35% in comparison to solution phase enzyme activity. This suggests that the matrix has a significant effect on the enzyme activity. Co-immobilization with FD results in further reduction of enzyme activity to an extent of 16% for FUAM. The drop in enzyme activity along with FD is a function of concentration of enzyme getting encapsulated. The residual urease activity in solution phase was found to be 90% and 40% at the end of 14 and 28 days, respectively. When Urease activity in UAM, FUAM was compared it was deduced that 23%, 40% drop takes place at the end of 14 days in comparison to solution phase activity (Fig. 3f). The comparison of sensing assay formulations, at the end of 14 days and 28 days shows that residual activity is observed following the pattern UAM > FUAM. When the slopes of enzyme activity are compared a significant drop in enzyme activity when present in alginate matrix than when present along with FD and alginate matrix. Compartmentalizing the fluorophore and enzymes can aid in reducing such an activity degradation due to presence of fluorophore.

## Methods

### Development and Characterization of Single Fluorophore/LBL assembled Ratiometric pH and Urea Biosensor

FITC-dextran (500 kDa) loaded Ca-alginate microspheres (FDAM) were prepared based on air driven atomization technique using an encapsulation unit or a droplet generator based on studies described by Joshi *et al*.^42, 43^. FDAM was prepared and optimized to form sizes in the range of 50 ± 10μm by varying parameters in the encapsulation unit (Nisco Var J3, Switzerland) like flow rate, concentration of sodium alginate (Sigma-Aldrich (India)) and calcium chloride Merck (Mumbai), distance from the nozzle to surface of liquid, air pressure etc. Briefly, FD (0.25–1 mg ml^−1^) (Sigma-Aldrich (India)) was dissolved in alginate (2%w/w) and sprayed through encapsulation unit at a flow rate (20 ml hour^−1^) and air pressure (75 mbar) into a well stirred CaCl_2_ solution (4% w/v). FITC-Dextran (FD) (0.25–1 mg ml^−1^) co-immobilized with urease (1–5 mg ml^−1^) (Sigma-Aldrich (India)) in alginate micro-carriers using similar instrumental parameters were developed to form FUAM. FDAM and FUAM were isolated and purified using deionized water in the form of triplicate cycles of centrifugation and stored at 4 °C. FDAM and FUAM were coated by layer-by-layer assembly using polyelectrolytes PAH (2 mg/ml) (Sigma-Aldrich (India)) and PSS (2 mg/ml) (Sigma-Aldrich (India)). Tris(bipyridine)ruthenium(II) chloride (RuBpy) (positively charged dye) (Sigma-Aldrich (India)) (1 mg/ml) was mixed with PSS to form ionic bonding between the two components. The resultant solution was dialyzed using 12–14 kDa dialysis membrane to remove unbound RuBpy. Dialyzed mixture was assembled upon PAH coated FDAM and FUAM to form FDAM-PAH-PSS-RuBpy-PAH (RDAM) and FUAM-PAH-PSS-RuBpy-PAH (RUAM). pH and urea sensing micro-carriers so formed were washed with deionized water by centrifugation and stored at 4 °C before using for further experiments.

The microcarriers FDAM, FUAM, RDAM and RUAM were characterized using optical microscopy (Nikon optical microscope), scanning electron microscopy (SEM) (Hitachi S-3400, Japan), confocal laser scanning microscopy (CLSM) (Olympus FluoView^TM^, Japan). Optical and SEM images of plain alginate microspheres (PAM), FDAM, FUAM, RDAM, RUAM were captured optical and SEM to study morphology, particle size, distribution and aggregation characteristics. Mean particle size and standard deviation was determined by measuring diameter of 100 particles for each formulation of sensors. FDAM, FUAM, RDAM, RUAM were examined using CLSM equipped with a krypton-argon laser. Distribution of FD and RuBpy within the alginate carriers were also studied using line scan measurements in CLSM. Success of LBL assembly was confirmed using zeta potential measurement after each coating using Brookhaven’s BIC PALS zeta potential analyzer, USA. Encapsulation efficiency of FD and urease was determined by estimating the amount of un-encapsulated FD/urease in supernatants using UV-visible spectroscopy either directly (482 nm) or by Bradford test (595 nm).

### Biosensing and Validation of pH and Urea Biosensors

FDAM and RDAM particles were exposed to buffers with different pH values in the range of 4–8. Fluorescence emission intensities at 520 nm or 520 nm and 610 nm, respectively were captured from fluorescent scans. Intensity ratios (I_520_/I_610_) were plotted against different pH values for ratiometric estimation of pH. FUAM and RUAM (100 μl) particles were exposed with urea concentrations in the range of 0–50 mM (10 μl) and volume made up to 1.5 ml with Milli Q water. Fluorescence emission scans were captured (λ_ex_ = 488 nm, λ_em1_ = 520 nm, λ_em2_ = 610 nm) to calculate intensity ratio (I_520_/I_610_) for different urea concentrations. Linearity, range and limit of detection for pH and urea sensors was determined. Inter-day reproducibility studies for RDAM and RUAM were performed for 3 consecutive days.

### Detection of pH and Urea in Urine Samples

Urine samples (n = 3) obtained for routine testing and kidney disease patients diagnosed with chronic kidney disease were procured from KEM hospital, Mumbai, India and Apex Kidney Care, Mumbai, India during January to December 2016 and April 2017, respectively. Necessary ethical clearances for this study were obtained from the institutional ethics committee (IEC-II Project No. EC/184/2013) of Indian Institute of Technology Bombay, KEM Hospital, Mumbai, India and Apex Kidney Care Mumbai, India. Informed consent was not needed as KEM Hospital pathological labs were used as per the ethical approval. All the experiments were performed in accordance with the guidelines and regulations for biomedical research on human participants as per Indian Council of Medical Research. pH detection using FDAM/RDAM was directly performed by exposing urine samples and referenced using a commercially available laboratory pH meter (Orion pH meter, USA) in triplicate. In case of urea measurement, urine samples were diluted to about 20 times and the resulting solution was used for spiking different concentrations of urea (0–100 mM). The spiked concentrations were then exposed to sensors (FUAM/RUAM), after necessary incubation to allow for enzymatic reaction the fluorescence scans were captured and pH values or urea concentrations determined using calibration curves of biosensors. Concentrations present in the urine were calculated after deducting the initial concentration before spiking the samples. The difference of concentration detected was used for calculation of percent recovery, which represents the accuracy of sensors in urine samples.

### Stability, Interference and Leaching Studies of pH and Urea Biosensors

FDAM, FUAM, RDAM, and RUAM were evaluated for storage stability over a period of one month. The biosensors were stored at 4 °C during storage. The slopes/relative sensitivity of sensing experiments were compared to evaluate the stability of biosensors over a period of time. Dye leaching was another parameter tested for evaluating the sensor stability. Urea sensing stability studies were carried out every week for a period of one month (0, 7^th^, 14^th^, 21^th^, 28^th^ days). FUAM was also subjected to interference study using patho-physiologically present clinical constituents like sodium (260 mM), potassium (100 mM), calcium (7.5 mM), ammonium (55 mM), chloride (250 mM), phosphate (40 mM), sulphate, albumin (80 mM), bilirubin (40 mM), glucose (5 mM), uric acid (100 µM), ascorbic acid (100 µM) and buffers so that the performance of urea sensor can be evaluated. Urea sensor performance in different buffers were evaluated in the physiological and pathological concentrations. The FDAM, FUAM, RDAM, and RUAM microparticles were subjected to leaching studies by collecting supernatants after centrifugation from sensor particles suspended in deionized water. 2 ml of supernatant solution was withdrawn with replacement. Supernatant solutions were characterized using fluorescence spectroscopy for FD/RuBpy leaching using fluorescence spectroscopy and Urease leaching using Bradford’s assay using UV-visible spectroscopy. Nessler’s reaction was used in order to measure the ammonium ions produced during urea catalytic reaction in presence of urease. Briefly, 0.2 M urea was exposed to urease solutions (1 mg ml^−1^) in 50 mM Tris Buffer (pH 7.3) with 10 min incubation. This reaction mixture (1 ml) was mixed with 1 ml Nessler’s reagent and diluted to 50 ml and absorbance measured at 405 nm using UV-visible spectroscopy. For immobilized urease systems like FUAM and RUAM were incubated with intermittent agitation when exposed to 0.2 M Urea solutions in 50 mM tris buffer (pH 7.3). An enzyme unit is defined as the amount of enzyme required to liberate the 1 µmol of ammonia/min in our test conditions (Tris buffer (pH 7.3), 0.2 M urea, and 10 min, at room temperature). The enzyme activity in formulations was compared over a period of one month. The biosensors were then exposed to urine samples by spiking different concentrations in the range of calibration curves and calculating the accuracy of estimation of spiked concentrations. In case of pH sensing, reference measurements were taken using a sensitive pH meter.

### Statistical analysis

Data are presented as mean ± standard deviation of three independent experiments (n = 3) for all sensing experiments. Statistical significance was determined by Student’s t-test (two-tail) between two groups.

## Conclusions

An accurate fluorescence based method for detection of pH and urea using FD (a single fluorophore) and FD-RuBpy combination (ratiometrically) has been demonstrated employing FD-alginate based microspheres. Co-immobilization of urease along with FD and susequnt application of LBL assembly can bring advanced features in urea biosensing in microcarriers of ratiometric determination. The findings suggest that the developed pH biosensor FDAM and RDAM show a detection capability in the range of pH units 6–8 and for urea biosensor FUAM and RUAM show sensing in the range of 0–50 mM. The analysis of biosensors (FDAM, RDAM, FUAM, RUAM) suggested that pH and urea detection is possible accurately with an accuracy of 95–104% in standard solutions. In urine samples, the accuracy of FDAM was equivalent to that of RDAM but the accuracy of RUAM was found to be better than FUAM. pH and urea biosensor compositions were evaluated and validated for reproducibility, response time, linearity, range, leaching and stability. The results indicate that proposed biosensors have great potential to be translated to produce disposable biosensors for point-of care measurement.

## Electronic supplementary material

Supplementary Information

## References

[1] Lee W-Y (2000). Sol-gel-derived thick-film conductometric biosensor for urea determination in serum. Anal. Chim. Acta.

[2] Chaudhari PS, Gokarna A, Kulkarni M, Karve MS, Bhoraskar SV (2005). Porous silicon as an entrapping matrix for the immobilization of urease. Sens. Actuators, B.

[3] Koncki R, Radomska A, Glab S (2000). Bioanalytical flow-injection system for control of hemodialysis adequacy. Anal. Chim. Acta.

[4] Soldatkin AP, Montoriol J, Sant W, Martelet C, Jaffrezic-Renault N (2003). A novel urea sensitive biosensor with extended dynamic range based on recombinant urease and ISFETs. Biosens. Bioelectron.

[5] Luo Y-C, Do J-S (2004). Urea biosensor based on PANi(urease)-Nafion®/Au composite electrode. Biosens. Bioelectron.

[6] Kovacs B, Nagy G, Dombi R, Toth K (2003). Optical biosensor for urea with improved response time. Biosens. Bioelectron.

[7] Ruedas-Rama MJ, Hall EA (2010). Analytical nanosphere sensors using quantum dot-enzyme conjugates for urea and creatinine. Anal. Chem. (Washington, DC, US).

[8] Tsai H-C, Doong R-A (2007). Preparation and characterization of urease-encapsulated biosensors in poly(vinyl alcohol)-modified silica sol-gel materials. Biosens. Bioelectron.

[9] Michalec M (2016). Optoelectronic detectors and flow analysis systems for determination of dialysate urea nitrogen. Sensors and Actuators B: Chemical.

[10] Li Z, Li L-J, Sun T, Liu L, Xie Z (2016). Benzimidazole-BODIPY as optical and fluorometric pH sensor. Dyes and Pigments.

[11] Tian M, Peng X, Fan J, Wang J, Sun S (2012). A fluorescent sensor for pH based on rhodamine fluorophore. Dyes and Pigments.

[12] Wu A, Duan L (2011). A near-infrared fluorescent sensor for H+ in aqueous solution and living cells. Turk J Chem.

[13] Safitri E, Heng LY, Ahmad M, Ling TL (2017). Fluorescence bioanalytical method for urea determination based on water soluble ZnS quantum dots. Sensors and Actuators B: Chemical.

[14] Wolfbeis OS, Posch HE (1986). Fibre optic fluorescing sensor for ammonia. Anal. Chim. Acta.

[15] Rhines TD, Arnold MA (1989). Fiber-optic biosensor for urea based on sensing of ammonia gas. Anal. Chim. Acta.

[16] Xie X, Suleiman AA, Guilbault GG (1991). Determination of urea in serum by a fiber-optic fluorescence biosensor. Talanta.

[17] Zhou Y (2016). FITC Doped Rattle-Type Silica Colloidal Particle-Based Ratiometric Fluorescent Sensor for Biosensing and Imaging of Superoxide Anion. ACS Applied Materials & Interfaces.

[18] Ramakrishnam Raju MV, Chandra Prakash E, Chang H-C, Lin H-C (2014). A facile ratiometric fluorescent chemodosimeter for hydrazine based on Ing–Manske hydrazinolysis and its applications in living cells. Dyes and Pigments.

[19] Maity D, Karthigeyan D, Kundu TK, Govindaraju T (2013). FRET-based rational strategy for ratiometric detection of Cu2+ and live cell imaging. Sensors and Actuators B: Chemical.

[20] Barker SLR, Kopelman R (1998). Development and Cellular Applications of Fiber Optic Nitric Oxide Sensors Based on a Gold-Adsorbed Fluorophore. Analytical Chemistry.

[21] Ueberfeld J, Walt DR (2004). Reversible Ratiometric Probe for Quantitative DNA Measurements. Analytical Chemistry.

[22] Jia C, Wu B, Liang J, Huang X, Yang XJ (2010). A colorimetric and ratiometric fluorescent chemosensor for fluoride based on proton transfer. Journal of fluorescence.

[23] Zeng X (2011). A colorimetric and ratiometric fluorescent probe for quantitative detection of GSH at physiologically relevant levels. Sensors and Actuators B: Chemical.

[24] Mahapatra AK (2011). A highly sensitive and selective ratiometric fluorescent probe based on conjugated donor–acceptor–donor constitution of 1,8-naphthyridine for Hg2+. Journal of Photochemistry and Photobiology A: Chemistry.

[25] Ma F, Sun M, Zhang K, Wang S (2015). A ratiometric fluorescence sensor for highly selective and sensitive detection of mercuric ion. Sensors and Actuators B: Chemical.

[26] Dai C, Yang CX, Yan XP (2015). Ratiometric Fluorescent Detection of Phosphate in Aqueous Solution Based on Near Infrared Fluorescent Silver Nanoclusters/Metal-Organic Shell Composite. Anal Chem.

[27] Gao F, Tang L, Dai L, Wang L (2007). A fluorescence ratiometric nano-pH sensor based on dual-fluorophore-doped silica nanoparticles. Spectrochimica Acta Part A: Molecular and Biomolecular Spectroscopy.

[28] Ke CY, Wu YT, Tseng WL (2015). Fluorescein-5-isothiocyanate-conjugated protein-directed synthesis of gold nanoclusters for fluorescent ratiometric sensing of an enzyme-substrate system. Biosensors &amp; bioelectronics.

[29] Liu YY, Wu M, Zhu LN, Feng XZ, Kong DM (2015). Colorimetric and Fluorescent Bimodal Ratiometric Probes for pH Sensing of Living Cells. Chemistry, an Asian journal.

[30] Hilderbrand SA, Kelly KA, Niedre M, Weissleder R (2008). Near Infrared Fluorescence-Based Bacteriophage Particles for Ratiometric pH Imaging. Bioconjugate Chemistry.

[31] Zhang Y, Guo S, Cheng S, Ji X, He Z (2017). Label-free silicon nanodots featured ratiometric fluorescent aptasensor for lysosomal imaging and pH measurement. Biosensors &amp; bioelectronics.

[32] Guha S, Shaw GK, Mitcham TM, Bouchard RR, Smith BD (2016). Croconaine rotaxane for acid activated photothermal heating and ratiometric photoacoustic imaging of acidic pH. Chemical Communications.

[33] Wu P, Hou X, Xu J-J, Chen H-Y (2016). Ratiometric fluorescence, electrochemiluminescence, and photoelectrochemical chemo/biosensing based on semiconductor quantum dots. Nanoscale.

[34] Duong HD, Rhee JI (2015). Development of a ratiometric fluorescent urea biosensor based on the urease immobilized onto the oxazine 170 perchlorate-ethyl cellulose membrane. Talanta.

[35] Tsai H-C, Doong R-A (2004). Simultaneous determination of renal clinical analytes in serum using hydrolase- and oxidase-encapsulated optical array biosensors. Anal. Biochem..

[36] Tsai H-C, Doong R-A (2005). Simultaneous determination of pH, urea, acetylcholine and heavy metals using array-based enzymatic optical biosensor. Biosens. Bioelectron.

[37] Kazakova LI, Shabarchina LI, Sukhorukov GB (2011). Co-encapsulation of enzyme and sensitive dye as a tool for fabrication of microcapsule based sensor for urea measuring. Physical Chemistry Chemical Physics.

[38] Chaudhary A, Srivastava R (2008). Glucose Sensing Using Competitive Binding Assay Co-Encapsulated in Uniform Sized Alginate Microspheres. Sens. Lett..

[39] Jayant RD, Srivastava R (2007). Dexamethasone Release from Uniform Sized Nanoengineered Alginate Microspheres. J. Biomed. Nanotechnol..

[40] Swati M, Srivastava R (2009). Stabilization of Sensing Assay within Polyelectrolyte-Coated Alginate Microspheres for Optical Urea Sensing. Anal. Lett..

[41] Jayant RD, McShane MJ, Srivastava R (2011). *In vitro* and *in vivo* evaluation of anti-inflammatory agents using nanoengineered alginate carriers: Towards localized implant inflammation suppression. Int. J. Pharm. (Amsterdam, Neth.).

[42] Joshi A, Keerthiprasad R, Jayant RD, Srivastava R (2010). Nano-in-micro alginate based hybrid particles. Carbohydr. Polym.

[43] Joshi A (2011). Multifunctional alginate microspheres for biosensing, drug delivery and magnetic resonance imaging. Acta Biomater.

[44] Chaudhari RD, Joshi AB, Srivastava R (2012). Uric acid biosensor based on chemiluminescence detection using a nano-micro hybrid matrix. Sens. Actuators, B.

[45] Chaudhari RD, Joshi AB, Pandya K, Srivastava R (2016). pH Based Urea Biosensing Using Fluorescein Isothiocyanate (FITC)-Dextran Encapsulated Micro-Carriers of Calcium Alginate. Sensor Letters.

[46] Swati M, Hase NK, Srivastava R (2010). Nanoengineered optical urea biosensor for estimating hemodialysis parameters in spent dialysate. Anal. Chim. Acta.

[47] Saxena A, Bhattacharya A, Kumar S, Epstein IR, Sahney R (2017). Biopolymer matrix for nano-encapsulation of urease - A model protein and its application in urea detection. Journal of colloid and interface science.

