# Supplementary material for: pH and Urea Estimation in Urine Samples using Single Fluorophore and Ratiometric Fluorescent Biosensors
Source: Sci Rep. 2017 Jul 19;7:5840. doi: 10.1038/s41598-017-06060-y (PMC5517509; doi:10.1038/s41598-017-06060-y)
Supplement: Supplementary file 1 — Supplementary Information [file 41598_2017_6060_MOESM1_ESM.doc]

**pH and Urea Estimation in Urine Samples using Single Fluorophore and Ratiometric Fluorescent Biosensors**

Rashmi D. Chaudhari, ^1^, Abhijeet B. Joshi, ^2^, Rohit Srivastava^1, *^

^1^ Department of Biosciences and Bioengineering, IIT Bombay, Powai, Mumbai, Maharashtra, Pin: 400 076, India

^2^ Centre for Biosciences and Biomedical Engineering, IIT Indore, Indore, Madhya Pradesh, Pin: 453 352, India

**Supplementary Information: Results**


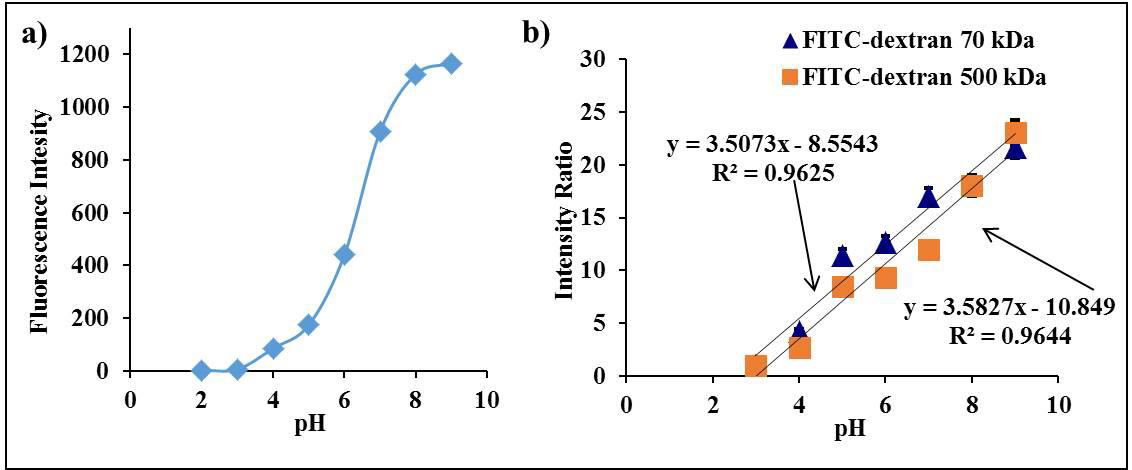


**Figure S1:** a) Typical fluorescence response of FD in response to different pH conditions and b) Effect of molecular weight of FD on pH response curves in FDAM.


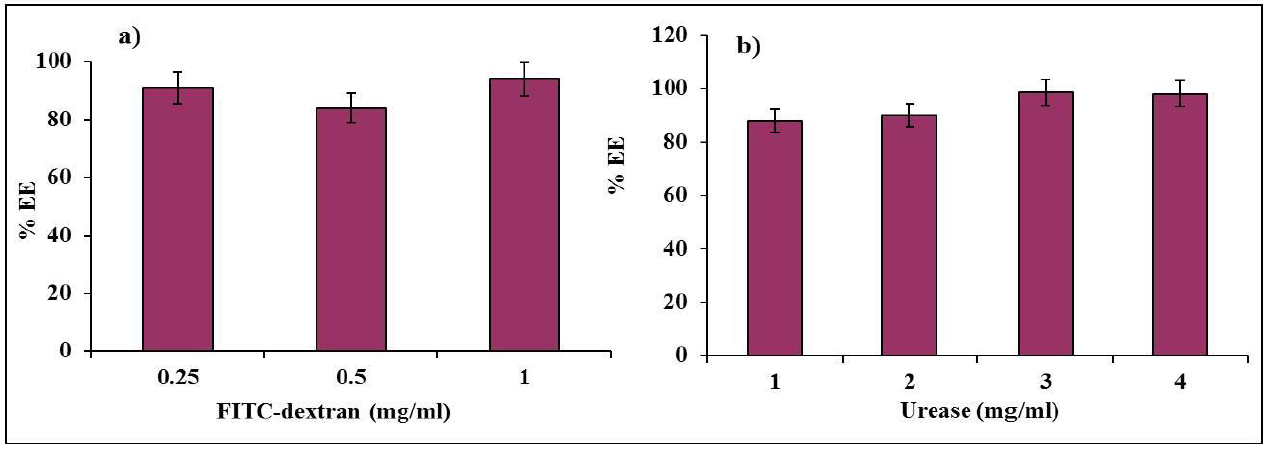


**Figure S2:** Encapsulation efficiency of FD and Urease in alginate microspheres


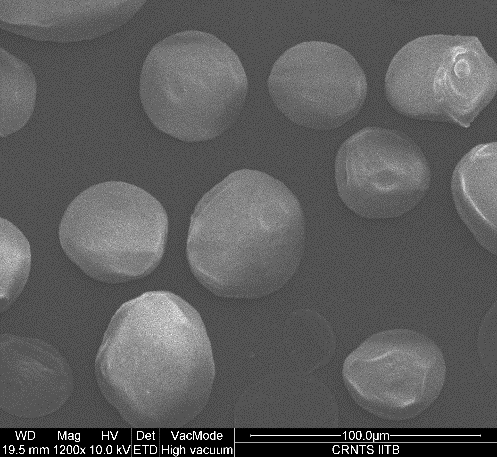

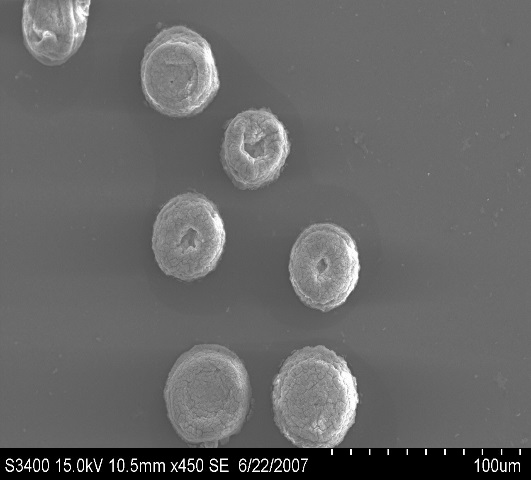


**Figure S3:** SEM images of FUAM and RUAM showing spherical morphology after loading of FD and Urease and rough surface after LBL self-assembly


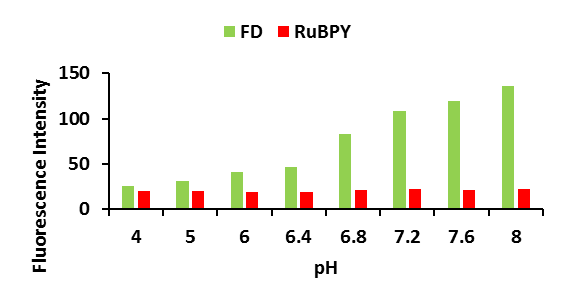


**a)**


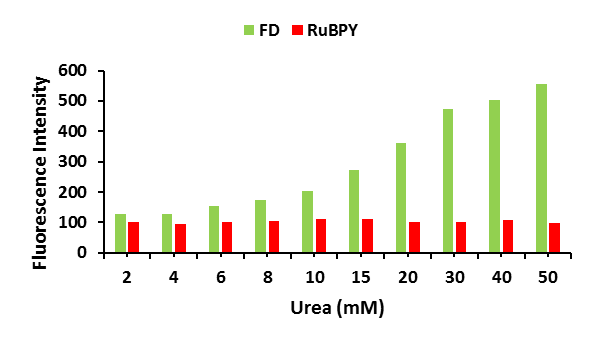


**b)**

**Figure S4:** pH and urea response curves for RDAM and RUAM indicating pH and urea responsiveness of FD (Indicator dye) with pH and urea stability of RuBPY (Reference dye)


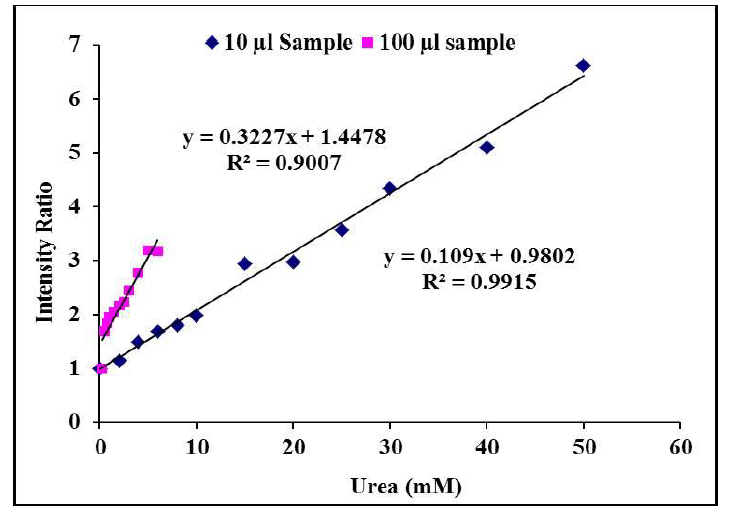


**Figure S5:** Effect to sample volumes (amount of urea concentration) exposed during sensing study.

**Table S1:** Effect of different buffers and their pH on urea sensor response in case of FUAM


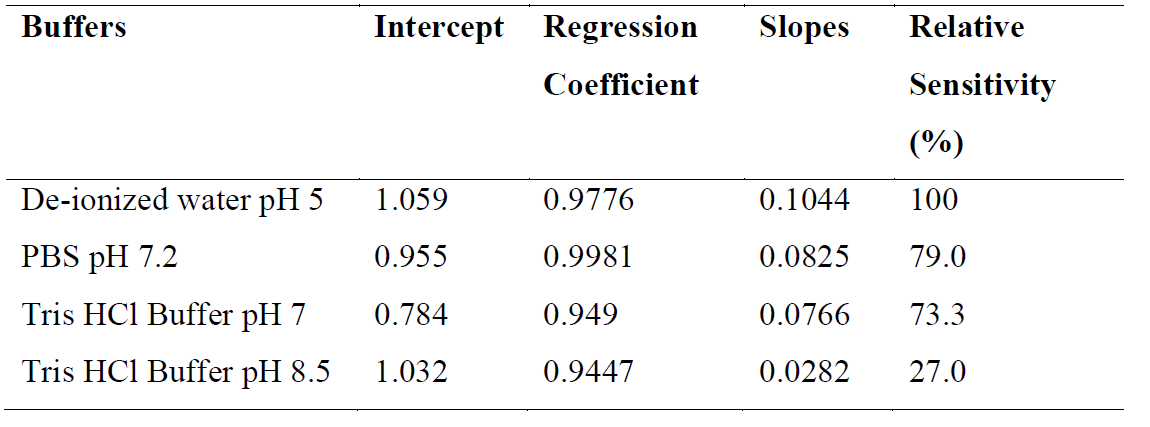


**Table S2: Accuracy Studies using standard samples of analytes (pH and urea)**

| **Sensor** | **Regression Equation** | **Regression Coefficient** |
| --- | --- | --- |
| FDAM | y = 0.9462x + 0.4508 | 0.999 |
| RDAM | y = 0.9327x + 0.4435 | 0.9879 |
| FUAM | y = 1.0096x - 0.2192 | 0.999 |
| RUAM | y = 1.0658x - 0.8915 | 1 |
